# Audio Quality Perception of Hearing-Impaired Listeners in Complex Acoustic Environments

**DOI:** 10.1177/23312165251374938

**Published:** 2025-10-23

**Authors:** Thomas Biberger, Stephan D. Ewert

**Affiliations:** 1 Department of Medical Physics and Acoustics and Cluster of Excellence Hearing4all, 597451 University of Oldenburg, Oldenburg, Germany

**Keywords:** audio quality perception, hearing loss, complex acoustic environments, psychoacoustic masking, distortion detection thresholds, binaural hearing

## Abstract

The effect of complex acoustic environments (CAEs), typically comprising target and interfering sound sources as well as room reflections, on the speech reception of hearing-impaired (HI) listeners has been examined in several studies. However, only little is known about audio quality perception of HI listeners in such CAEs. Thus, this study assessed detection thresholds and suprathreshold audio quality ratings of listeners with very mild and moderate hearing loss (HL) for several distortions applied to speech and pink noise: nonlinear saturation, spectral ripples, level differences, and spatial position offsets. The stimuli were presented in acoustical scenes that differ in their complexity by manipulating room size in conjunction with reverberation time, and the number and spatial position of interfering sound sources. The strongest differences between listeners with very mild and moderate HL were observed in the presence of interfering sounds. In such situations, listeners with moderate HL had consistently higher distortion detection thresholds than listeners with very mild HL. Moreover, they rated audio quality lower for the masked than for the unmasked distorted targets, indicating difficulties in separating the target from the maskers. Significant correlations were found between the listeners’ pure tone average (PTA) and distortion detection thresholds in situations with maskers. Thus, PTAs seem to be a suitable predictor for distortion thresholds of HI listeners in CAEs. The effect of reverberation strongly depended on the target (speech or pink noise) and the type of distortions.

## Introduction

In everyday life, target sounds are often transmitted via electroacoustic systems, e.g., a TV set, sound reproduction system, or earphones, typically involving audio-signal processing, which may introduce perceptible distortions, affecting perceived audio quality. In this context, two aspects are particularly important: (a) Detectability thresholds for the distortions, and (b) perceived audio quality for suprathreshold distortions. Previous studies examined these aspects mostly under (nearly) optimal conditions without interfering sound sources and reverberation (e.g., Emiya et al., 2011; Fleßner et al., 2017, 2019; ISO/IEC, 11172.3, 1993; Moore & Tan, 2003; Tan et al., 2003; Van Buuren et al., 1999). Given that the distorted sounds might be played back in reverberant rooms or in the presence of other interfering sound sources, Biberger and Ewert (2022) investigated the consequences of reverberation as well as the number and spatial distribution of interfering sound sources on distortion detection thresholds and suprathreshold quality ratings. They found significant effects of maskers and reverberation on distortion thresholds and suprathreshold quality ratings. For example, detection thresholds were significantly higher when the distorted signal was presented with than without maskers. Overall, they demonstrated that the perceived audio quality of young normal-hearing (YNH) listeners may depend on whether the distorted signals were presented in optimal conditions without reverberation and masking sounds or in more ecologically relevant complex acoustic environments (CAEs, e.g., Keidser et al., 2020).

Young listeners with healthy auditory systems are very good at separating the desired from the masking sound sources by using level and spatial cues (Biberger & Ewert, 2019; Cherry, 1953; Fichna et al., 2021; Plomp, 1976) or voice/timbre characteristics (e.g., Brungart, 2001; Iverson, 1995). Elderly listeners with sensorineural hearing loss (HL), typically caused by damage to the hair cells in the cochlea or pathology of the auditory nerve, often have more difficulties in such complex scenes (e.g., Buchholz et al., 2023; Humes, 1991; Mansour et al., 2021; Plomp, 1978). Such sensorineural HL is often associated with a reduced frequency selectivity and loss of compressive nonlinearity on the basilar membrane (BM), which can be expected to influence auditory cues required to separate the target from the masker signals. Loudness perception of hearing-impaired listeners with cochlear damage is strongly impaired as they typically show increased absolute thresholds and often show much steeper growth of loudness (e.g., Brand & Hohmann, 2001; Moore, 1998; Pieper et al., 2018; Steinberg & Gardner, 1937) with increasing sound level (mainly due to the loss of compression on the BM). This effectively reduces the dynamic range of sound levels (from audible to uncomfortable) for HI listeners. The reduced frequency selectivity has also effects on timbre perception—depending on spectral and temporal sound properties (e.g., Emiroglu & Kollmeier, 2008; Handel, 1995). Fewer details about the spectral composition of a sound are encoded by broader filters, which makes it more difficult to discriminate sounds based on spectral properties. While temporal fine structure (TFS) or pitch cues can be helpful for normal hearing (NH) listeners to separate the target from masking fluctuating sounds (e.g., Moore, 2008, 2021) or improve speech perception (Hopkins et al., 2008), only slight improvements were observed for HI listeners. It was hypothesized that HL affects the availability of TFS cues, in line with effects of HL and age on the use of TFS cues reported in Paraouty et al. (2016), Kortlang et al. (2016), and Ewert et al. (2018). Besides the above-described monaural cues, binaural (spatial) cues are important to separate the target from the masker. While HI listeners generally perform worse in spatial-hearing experiments than NH listeners (for an overview, see Akeroyd, 2014), situations where the target was presented with interfering sound sources are particularly critical (e.g., Best et al., 2011; Häusler et al., 1983; Lorenzi et al., 1999a, 1999b).

From the above-outlined perceptual consequences of sensorineural HL, it seems not surprising that in the context of audio quality perception, HI listeners perceive distorted signals differently from NH listeners, even in non-CAEs, as observed in Lawson and Chial (1982), Kates and Kozma-Spytek (1994), Kozma-Spytek et al. (1996), and Stelmachowicz et al. (1999). Arehart et al. (2007, 2010) found significantly different ratings in speech quality judgements for effects of noise, clipping-distortion, and distortions associated with hearing aid processing in NH and HI listeners. In Arehart et al. (2011), significant differences between the music quality ratings of NH and HI were only found for quantization and peak clipping, while no significant differences between both groups were found for other distortion types.

For CAEs, it remains unclear how HI listeners perceive distortions in the presence of late reverberation and interfering sound sources. Knowledge about audio quality perception in such scenes is relevant for hearing device development (e.g., when aiming at acoustical transparency) as well as for the development of instrumental measures for audio quality.

Therefore, this study examined the effect of acoustic scene complexity on the HI listeners’ distortion detection thresholds (audibility) and suprathreshold quality ratings. Two groups of listeners with either very mild or moderate HL took part in the experiments to assess different levels of sensorineural HL. To control for effects of audibility, HL was partly compensated based on the audiogram using the linear amplification strategy NAL-R (Byrne & Dillon, 1986), resulting in a similar sensation level across both HI groups. To avoid age-related effects on suprathreshold auditory processing or cognitive factors, as they have often been reported for challenging acoustic scenarios (e.g., Carroll et al., 2016; Pichora-Fuller et al., 1995), both listener groups in this study were matched in age.

Based on Biberger and Ewert (2022), four types of distortions which are associated with different auditory cues were applied to the target signals: (a) spectral ripples (linear distortions), (b) a saturating, instantaneous nonlinearity (nonlinear distortions), (c) differences in the target sound-source intensity, and (d) variation of the spatial position of the target (azimuthal direction of 0°, 8°, and 30° relative to the listener's frontal direction). The target was either speech or pink noise to probe differences in the spectro-temporal characteristics. Given the importance of speech in daily life, it was used in all experiments of this study, while pink noise, more representative of environmental background noise, was only used in a subset of experiments. The International Speech Test Signal (ISTS; Holube et al., 2010) and a pop music excerpt, reflecting typical (masking) sounds in CAEs, were used as maskers.

In the first experiment, distortion detection thresholds, related to the audibility of distortions, were measured in three room configurations: with (i) mild, (ii) moderate reverberation, and (iii) with mild reverberation and two spatially separated maskers. In the second experiment, suprathreshold audio quality ratings for signals with low and high distortion levels were obtained from acoustic scenes with different reverberation times and various masker configurations, where the number and spatial position of the maskers were changed. The following research questions were addressed in this study: (a) What is the effect of reverberation and interfering sound sources on the distortion detection thresholds and suprathreshold quality ratings of listeners with very mild and moderate HL? (b) Can audiogram information be used as a predictor for distortion detection thresholds or quality ratings? (c) Is the listeners’ individual performance in distortion detection thresholds a predictor for suprathreshold quality ratings?

## Methods

### Listeners

**Figure 1.**
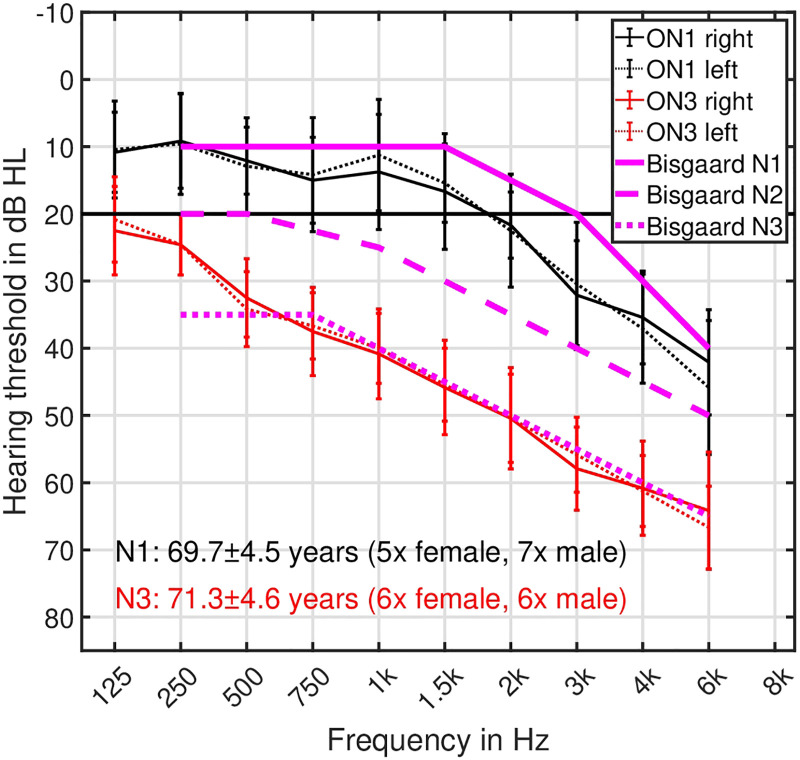
Audiograms (air conductance) show the hearing thresholds in dB HL for frequencies ranging between 125 Hz and 6 kHz. Black and red symbols/lines indicate ON1 and ON3 listeners, whereas solid and dotted lines represent the listener's right and left ears. Prototypical N1, N2, and N3 audiogram curves proposed in Bisgaard et al. (2010) are shown as solid, dashed, and dotted magenta lines. The error bars represent one standard deviation.

Twelve older listeners with very mild HL (5 female, 7 male, mean age: 69.7 years), matching the N1 (“very mild” HL) Bisgaard category (Bisgaard et al., 2010) in frequencies ranging from 250 Hz to 6 kHz, and 12 older listeners with moderate HL (6 female, 6 male, age: 71.3 years), matching the N3 (“moderate” HL) Bisgaard category in frequencies ranging from 250 Hz to 6 kHz took part in this study. The listeners were assigned to the Bisgaard category for which the measured audiogram data showed the smallest RMSE-based difference, resulting in either the N1 or N3 categories. In the following, the older N1 and N3 listeners are denoted as ON1 and ON3, respectively. The level differences between the left and right ears' audiograms were not higher than 10 dB, referring to a symmetrical bilateral HL. The ON1 listeners were not used to wear hearing aids, while all ON3 listeners were used to wear hearing aids. The averaged audiograms for the left and right ears of the ON1 and ON3 listeners are shown in Figure 1. Most of the listeners had previously participated in hearing-related experiments. All listeners received financial compensation for their participation.

### Stimuli

#### Target and Masker Signals

The same German speech (spoken language) and pink-noise stimuli as used in Fleßner et al. (2019) and Biberger and Ewert (2022), having different spectro-temporal properties, were applied as targets. The speech stimulus was spoken by a female speaker and shows slow amplitude modulations (5-Hz range) and a relatively narrowband spectrum. The pink noise was a stationary stimulus with a broadband spectrum ranging from 10 to about 22 kHz, and covering the entire audible frequency range. All target stimuli had a duration of 2 s for the audio quality rating experiments, while in the distortion detection and discrimination experiments, the 2-s speech target was divided into 1-s segments which were randomly selected per trial.

Since typical communication situations often contain various different sound sources, target and masker sounds were selected that differ significantly in their signal properties. In addition to using a pop-music excerpt taken from Fleßner et al. (2019) as a masker, the male-transformed version of the ISTS speech signal (Holube et al., 2010), as applied in Schubotz et al. (2016) and Ewert et al. (2017), was used as a masker for the female target speaker. This combination results in more pronounced differences in acoustic features (e.g., pitch and timbre) than a female masker paired with a female target. The same maskers were also used in the audio quality study of Biberger and Ewert (2022) with NH listeners, enabling a comparison to their data. ISTS is nonsense speech generated from six different female speakers in different languages (American-English, Arabic, Mandarin, French, German, and Spanish). The music signal includes multiple instruments and vocals, with a rather broadband spectrum. In the audio quality rating experiments, the maskers had a duration of 2.5 s and started 0.5 s before the target onset, whereas the target and masker started simultaneously in the distortion detection and discrimination experiments. Raised-cosine ramps of 10 ms were applied to the masker and target stimuli. All signals were convolved with binaural room impulse responses (BRIRs) to define their spatial position and to simulate room reverberation (see Sec. Rooms and Masker Configurations).

#### Target Stimulus Distortions

Identical to Biberger and Ewert (2022), the target stimuli were subjected to four different types of distortions: spectral ripples, nonlinear saturation, intensity-based, and spatial:

Spectral ripples (linear distortions) were introduced as described in Fleßner et al. (2019), using sinusoidal modulation of the spectral envelope. Ten periods of the spectral sinusoidal modulation were applied between 50 Hz and 16 kHz, with equidistant spacing on a logarithmic frequency axis, corresponding to about 1.2 spectral ripples per octave. The spectral modulation depth (peak-to-valley ratio in dB) was adjusted to change the amount of distortion.

Nonlinear distortions caused by a simple instantaneous symmetric saturating input-output (I/O) characteristic (referred to as nonlinear saturation) simulated signal distortions caused by, e.g., large displacements of the loudspeaker diaphragm at high signal levels. The I/O characteristic was implemented as $y(t)=x(t)-\alpha \cdot (x(t){)}^{3}$ , where *x*(*t*) and *y*(*t*) are input and output signals, respectively. The factor α weights the cubic term relative to *x*(*t*), and thus controls the nonlinearity of the I/O characteristic. Input values were limited to the range $\pm \sqrt{\frac{1}{3\cdot \alpha }}$ where the nonlinear I/O characteristic completely saturates (soft clipping). This saturating I/O function resulted in pronounced harmonic distortions at higher signal levels, typically occurring at signal onsets and transients. These additionally introduced frequency components likely provided spectral or amplitude modulation cues to the listeners.

Intensity-based distortions were introduced by adjusting the overall sound level in dB relative to the level of the reference signal. In contrast to spectral ripples and nonlinear saturation, no spectral amplitude modulation cues were introduced.

Spatial (binaural) distortions were introduced by changing the azimuth location of the target using the appropriate BRIRs. The reference target was always presented in front (0° azimuth) of the listeners, while the spatially distorted target was shifted to the right side (relative to the viewing direction of the listener). The minimum audible angles measured in the discrimination experiment are referred to as azimuth JNDs.

Anchor signals were generated by applying a 3.5-kHz low-pass filter, nonlinear saturation, and spatial distortion to the reference signals. The nonlinear saturation (α_speech_ = .55, α_noise_ = .55) and spatial distortion (position at 40° azimuth) in the anchor were more pronounced than the distortions applied in the other stimuli of this study.

For the detection experiment, the distortion level was adjusted during the experiment according to the listener's response (see Sec. Apparatus, Procedure, and Statistical Analysis), while for the quality rating experiments, distortions were applied at two different levels, denoted as low and high distortions, using the parameters provided in Table 1. The amounts of low and high distortions were based on a pilot experiment with two ON1 and two ON3 listeners and differed in 8 out of 16 cases from the values used in Biberger and Ewert (2022). For nonlinear saturation, Table 1 provides values for the dimensionless parameter α and the maximum total harmonic distortion (THD) for the peak value of the reference signals in percent.

**Table 1.** Experimental Parameters (and Units) Controlling the Amount of Distortions (Columns) in the Detection and Discrimination Experiments and for the Quality Rating Experiments (Low/High).

	Spectral ripples (peak-to-valley ratio in dB)	Nonlinear saturation (dimensionless parameter α)	Intensity (ΔdB re reference)	Spatial (Δ azimuth ° re reference)
Detection and discrimination experiments				
Starting value	30	0.82 (35.6)	8	28
Initial step size	5	0.2	2	4
Minimum step size	1.5	0.035	0.4	0.5
Suprathreshold quality ratings				
Speech	12/18	0.18 (22.1)/0.45 (31.4)	1.5/4.5	8°/30°
	*12/18*	*0.11 (15.3)/0.175 (21.4)*	*1.5/4*	*4°/30°*
Noise	2.5/5	0.22 (24.5)/0.44 (31.2)	1/4	8°/30°
	*2.5/5*	*0.18 (22.1)/0.37 (29.7)*	*1/4*	*4°/30°*

Parameters that are italicized refer to values used in Biberger and Ewert (2022) for YNH listeners. For a better representation of the amount of nonlinear distortion (second column), the THD@peak-values, given in percent, are provided in parentheses in addition to the dimensionless parameter α.

#### Rooms and Masker Configurations

Two room conditions were realized using headphone auralization and BRIRs generated by the room acoustics simulator (RAZR; Kirsch et al., 2023; Wendt et al., 2014). RAZR calculates early reflections up to the third order using the image source model (Allen & Berkley, 1979), while later reflections were calculated by a feedback delay network (Jot & Chaigne, 1991). An assessment of various common room acoustical parameters and subjective ratings of perceived room acoustical attributes showed a good correspondence between simulated and real rooms (see Blau et al., 2021; Brinkmann et al., 2019; Stärz et al., 2022; Wendt et al., 2014).

The digital twin of the living room lab (LRL), representing a typical German living room, has been developed by Schütze et al. (2021, 2025) at the University of Oldenburg, aiming at carrying out psychoacoustic and speech intelligibility experiments in ecologically valid scenes. The LRL was one of the acoustical scenes suggested by Van de Par et al. (2022) for ecologically valid hearing research and thus used in this study. The LRL has an average reverberation time (T60) of 0.5 s and dimensions of 4.97 × 3.78 × 2.71 m^3^ (length × width × height), resulting in a room volume of 51 m^3^. In addition to the LRL with mild reverberation, a large room (Large) with dimensions of 7.5 × 4.52 × 3 m^3^ (∼100 m^3^) and an average T60 of 1.5 s was used for the representation of environments with pronounced reverberation. This large room is identical to the one used in Biberger and Ewert (2022).

The target and masker sources were convolved with the BRIRs such that they were placed on each of the positions as indicated in Figure 4 in Van de Par et al. (2022) for the receiver (R1), target (S-TV), left (S4), and right (S5) maskers. The target was always presented in front of the receiver at 0° azimuth (with the exception of experiments on binaural distortions), and the target–receiver distance was 2.55 m and 2 m in the LRL and Large room, respectively. These distances were chosen to represent a typical distance between the receiver and the sound-emitting device, e.g., a TV. Different masker configurations were only examined in the LRL, where the 1M_sep_ condition refers to the situation where the masker was spatially separated by −45° azimuth from the target. In conditions 2M_co_, and 2M_sep_, the maskers were either presented co-located to the target (target and masker sources at position S-TV) or spatially separated by ±45° azimuth (S4 and S5) from the target. In the Large room the maskers had the same distance as the target (2 m), while in the LRL the receiver and maskers had a distance of 1.59 m, different to that of the target. During the stimulus generation for the LRL, a level reduction was applied so that target and masker sources had approximately the same level according to a receiver distance of about 2.55 m, which makes this setup more comparable to the setup used in Biberger and Ewert (2022). In the separated conditions, the masker to the left was always the ISTS speech signal, while the masker to the right was always the pop music excerpt. The direct-to-reverberant ratios between target and receiver (DRR_T_), between left masker and receiver (DRR_ML_), and between right masker and receiver (DRR_MR_) are given in Table 2 for all two rooms.

**Table 2.** Room Acoustical Properties of the Two Different Rooms.

Room	Volume (m^3^)	T60 (s)	DRR_T,0°_ (dB)	DRR_T,4°_ (dB)	DRR_T,30°_ in (dB)	DRR_ML_ in (dB)	DRR_MR_ in (dB)
LRL	51	0.5	−0.6/−0.7	−1.1/0.1	−6.4/4.2	6.2/−10.5	−10.5/6.1
Large	100	1.5	−6.7/−6.3	−7.8/−5.5	−13.5/−3.7	−3.1/−15.6	−16.6/−2.8

The third column is the reverberation time T60 in seconds. All DRRs are provided for the left/right ear. DRR_T,0°_ refers to the target and the co-located maskers placed at 0° azimuth, while DRR_T,4°_ and DRR_T,30°_ refer to the target source positions at 4° and 30° azimuth (spatial distortions). DRR_ML_ and DRR_MR_ refer to the spatially separated masker on the left (ISTS) and right (pop music), respectively. The values of DRR_T,0°_, DRR_T,4°_, and DRR_T,30°_ were calculated from the receiver-target-source BRIRs. DRR_ML_ and DRR_MR_ were calculated from the receiver-left-masker and receiver-right-masker BRIRs.

The receiver–target–masker positions were asymmetrically arranged in both rooms. Such an asymmetric arrangement in the room is more likely to occur in daily life than an unnatural, completely symmetrical arrangement and results in small long-term differences between the ears caused by early reflections, while no such differences are present for the direct sound. In the LRL, receiver, target, and masker sources were positioned at a height of 1.05, 1.10, and 1.02 m above the floor, while in the Large room, both receiver and target sources were positioned at a height of 1.7 m above the floor.

### Apparatus, Procedure, and Statistical Analysis

The stimuli were presented via Sennheiser HD650 headphones to the listeners, while seated in a double-walled, sound-attenuated booth. The stimuli were calibrated with a Bruel & Kjaer artificial ear (B&K Type 4153), and the transfer function of the headphones was digitally equalized to obtain a flat frequency response, in the same way as in Biberger and Ewert (2022).^1^ The level of the reference and masker signals at 0° in the LRL condition was 63 dB sound pressure level (SPL). Therefore, the masker configurations where the target was presented with two maskers at 0° had a signal-to-noise ratio (SNR) of about −3 dB. Depending on the reverberation time of the simulated room and the number of maskers, the overall level could reach up to about 68.5 SPL. Subjects were free to either respond via a touchscreen or mouse input. All audio files had a sampling rate of 44.1 kHz.

The measurement always started with collecting the (pure-tone) audiogram. Based on the audiogram data, the linear HL compensation strategy NAL-R (Byrne & Dillon, 1986) was calculated for each participant to ensure a sufficient audibility of the presented sound signals. In the next step, the participants adjusted the highest possible sound level to a comfortable level by selecting between −5 dB (level reduction: NAL-R-based level minus 5 dB), 0 dB (NAL-R-based level), and +5 dB (level increase: NAL-R-based level plus 5 dB). In the experiments, level differences between the sounds with the highest and lowest level were only about 5.5 dB, so that the sound signal with the lowest level is still expected to be sufficiently audible. Fourteen listeners selected a level reduction of 5 dB, eight listeners kept the NAL-R-based level, and two listeners selected an additional 5-dB level gain. NAL-R was used instead of the more recent nonlinear strategies, such as NAL-NL2, to avoid the nonlinear and time-variant effects of the HL compensation strategy, which could interact with the desired audio quality distortions. Moreover, since the largest overall level difference caused by the varying room acoustic scenarios as reported above was only about 5.5 dB, NAL-R appears to be an adequate compensation strategy to ensure the audibility of the stimuli. However, the present results may not fully generalize to everyday experiences of hearing aid users with nonlinear compensation strategies.

In the subsequent first experiment, a three-alternative, forced-choice (3-AFC) procedure was used to determine distortion detection and discrimination thresholds. For the sake of simplicity, the term “detection thresholds” is also used in the following for discrimination thresholds. Three intervals were presented, and listeners had to identify the randomly chosen interval containing the distorted speech (target). The strength of the distortion was varied according to a 1-up, 2-down procedure for estimating the 70.7% correct point of the psychometric function (Levitt, 1971). Stimuli in each trial were separated by 300-ms silent intervals. The initial and minimum step sizes used in the experiments are provided in Table 1. After the minimum step size was reached, six reversals were measured, from which the mean threshold was calculated. The final threshold was the mean of the estimates from two measurement runs (test–retest). All measurements were performed using the AFC framework (Ewert, 2013). The order of presentation of distortions was Latin-Square balanced, while the order of the room conditions, LRL, LRL with two spatially separated maskers (LRL,2M_sep_), and Large, was randomized, which is identical to Biberger and Ewert (2022). Prior to the actual measurement, listeners performed a training run for each type of distortion. The room selected for the training had a similar T60 as LRL and a somewhat different room size (see room Small in Biberger & Ewert, 2022).

For the suprathreshold audio-quality ratings in experiment 2, distorted speech and noise were used as the target. A measurement procedure applied in previous studies, Fleßner et al. (2017, 2019) and Biberger and Ewert (2022), was used, similar to the Multiple Stimulus Test with Hidden Reference and Anchor (MUSHRA, ITU-R, 2014). Listeners had to rate quality differences between several distorted targets, also denoted as test signals, and a given (unprocessed) reference target, by using a numerical rating scale ranging from 0 (“very strong difference”) to 100 (“no difference”). To ensure that listeners used the full range of the rating scale and to test the reliability of the listeners’ ratings, a hidden reference (without any distortions) and a strongly distorted anchor signal were included. The audio signals were played in a loop, and the listeners could listen as long as they wished. Listeners could also switch between the different test signals at any time, in which case the audio restarted at the beginning. The quality rating experiment was divided into two sub-experiments: In the first sub-experiment *Effect of room* the listeners rated audio quality for distorted speech and pink noise targets randomly presented in the LRL and Large room. In the second sub-experiment *Effect of masker*, the listeners rated distorted speech targets for different configurations of interfering maskers in the LRL room. After listeners finished the sub-experiment *Effect of masker*, they were asked to rate their ability in separating the speech targets from the interfering maskers (“How difficult was it to separate the target from the masker?”) by using a Likert-scale with five levels: −2: *very easy*, −1: *rather easy*, 0: *neutral*, +1: *rather difficult*, +2: *very difficult*. Prior to the actual measurement phase in each of the sub-experiments, a training run to familiarize the participants with the procedure was performed. For each of the quality rating experiments, listeners performed a test and a retest.

The entire measurement was divided into three sessions with 90 min duration each: In the first session, the audiogram was measured, followed by adjusting the comfortable level before the distortion detection thresholds for two out of four distortions were measured. In the second session, distortion detection thresholds for the remaining two distortions were measured before the listeners rated audio quality in the sub-experiment *Effect of room* (test). Only suprathreshold audio quality ratings were measured in the third session, where the participants started with the sub-experiment *Effect of masker* (test and retest), followed by the retest of the sub-experiment *Effect of room*. At the beginning of each session, the participants received a written instruction of the experiment. After reading the instructions, participants were asked by the supervisor of the experiment whether they had understood the instructions and if they had any questions. In the next step, the participants completed a training session and were then asked by the supervisor whether they were able to perform the experiment without any problems. All participants who had no problems with the training were then admitted to the main experiment.

For statistical analysis, mixed-design analysis of variance (ANOVA) was applied using IBM SPSS. Greenhouse–Geisser correction was applied if sphericity was violated. Bonferroni correction was applied in the post-hoc pairwise comparisons. For the analysis of the between-subjects effects based on HL, the Box-Cox transformation (BTC; Box & Cox, 1964) was applied to the data when necessary to stabilize the variances between groups.

## Results

**Figure 2.**
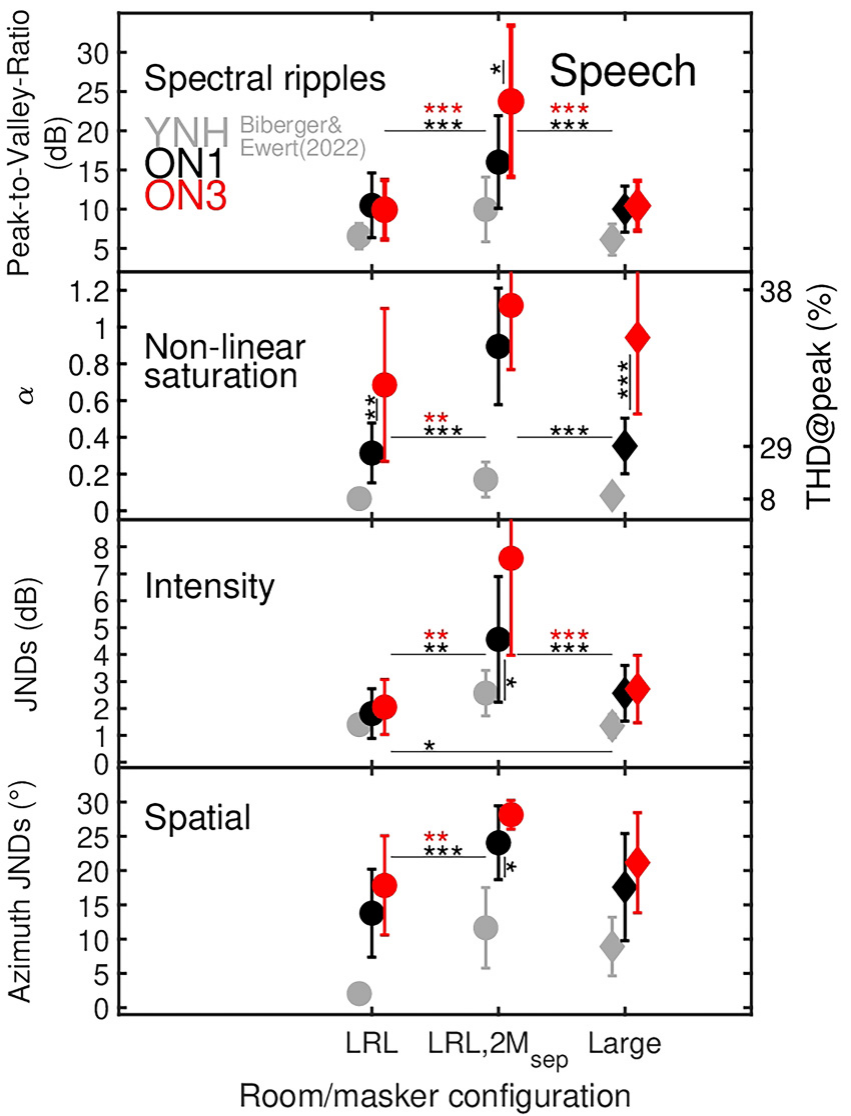
The panels show detection thresholds as black and red closed symbols for ON1 and ON3 listeners for the four types of distortions in the speech target presented with mild reverberation (LRL), with mild reverberation and two maskers (LRL,2M_sep_), and with moderate reverberation (Large). The rooms are represented by circles and diamonds, respectively. The error bars represent one standard deviation. Here and in the following figures, statistically significant post-hoc pair-wise comparisons based on levels of 0.05, 0.01, and 0.001 are indicated by *, **, and ***, respectively. Significant differences between room conditions are shown by horizontally aligned bars and symbols, while differences between listener groups are indicated by vertically aligned bars and symbols. With the exception of intensity distortions in LRL, statistically significant pair-wise comparisons between YNH and ON1/ON3 were consistently found for all types of distortion and all room/masker configurations. For improved readability, these differences are not shown in the figure. For nonlinear saturation, in addition to the dimensionless parameter α, the right *y*-axis provides the THD for the peak value (THD@peak) in percent.

**Figure 3.**
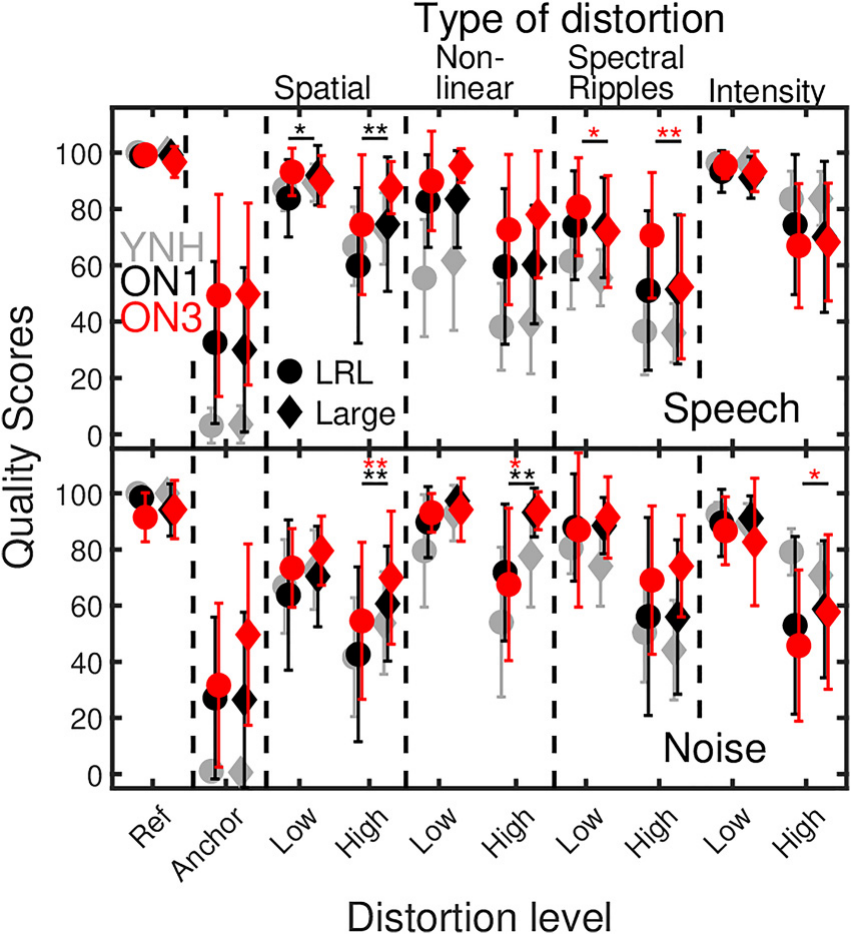
The upper and lower panels show suprathreshold audio quality ratings of ON1 (black filled symbols) and ON3 (red filled symbols) listeners for speech and noise. The ordinate represents quality scores ranging from 0 (“very strong difference”) to 100 (“no difference”). The abscissa represents the hidden reference, anchor, and type of distortion with low and high levels. The LRL and large room are represented by circles and diamonds, respectively. The error bars represent one standard deviation. Statistically significant pairwise comparisons between rooms are indicated by the asterisks. For comparison reasons, quality ratings of YNH listeners from Biberger and Ewert (2022) are replotted as gray symbols.

**Figure 4.**
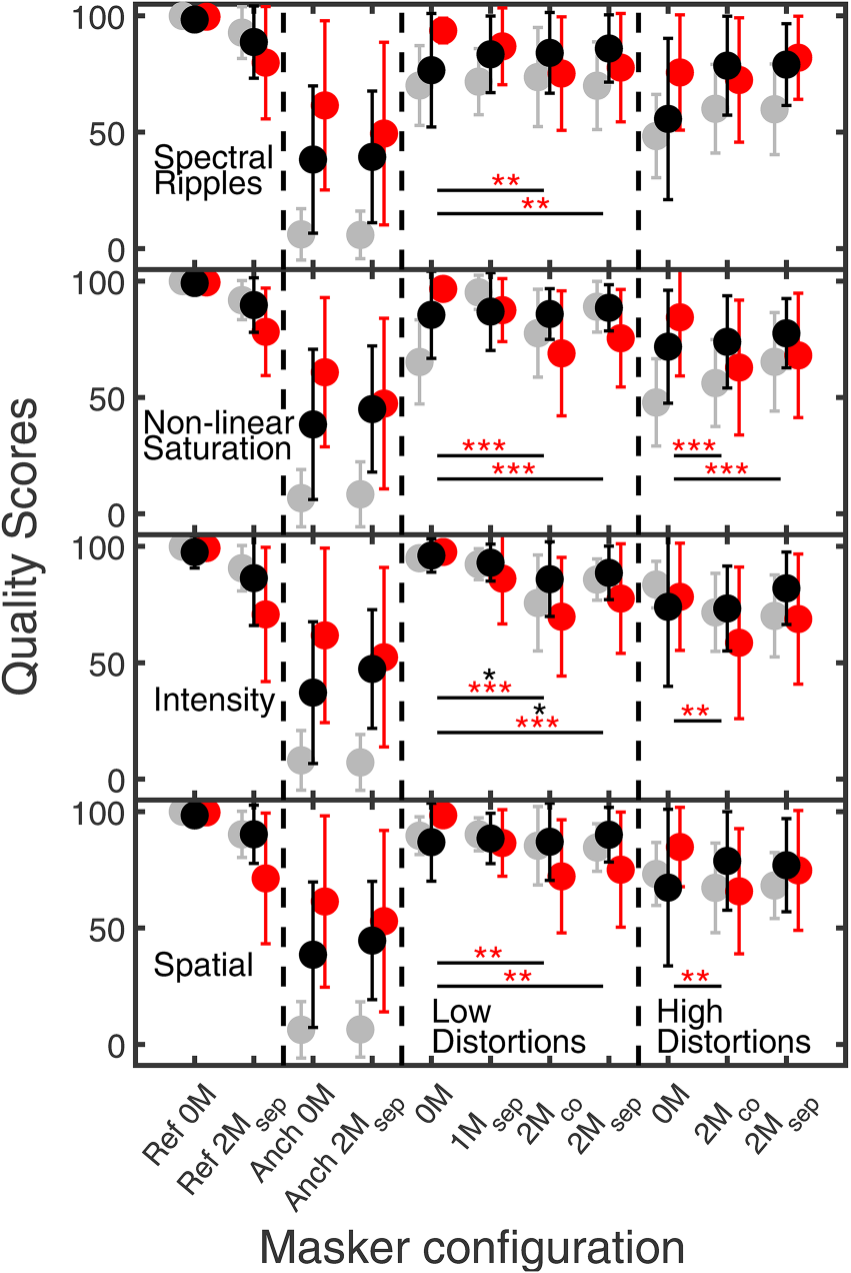
The four panels show quality ratings of ON1 and ON3 listeners, indicated by black and red closed symbols, for the speech target with spectral ripples, intensity distortion, nonlinear saturation, and spatial distortions in the LRL as a function of the masker configuration. Ref and Anch refer to the hidden reference and the anchor signals. 0M indicates no masker. In the configuration 1M_sep_, the ISTS masker was presented at −45° azimuth relative to the viewing direction of the listener. In configurations 2M_co_ and 2M_sep_ the ISTS and music maskers were presented at 0° and ±45° azimuth, respectively. Gray-filled circles represent quality ratings of YNH listeners from Biberger and Ewert (2022). The error bars represent one standard deviation.

**Figure 5.**
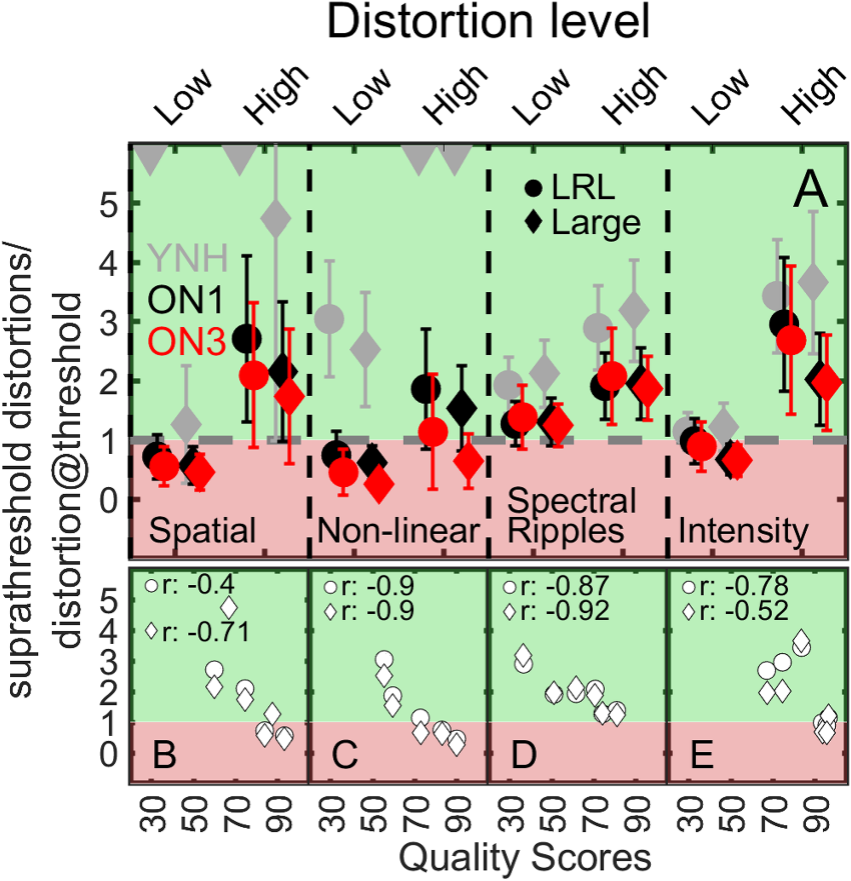
The Panel A shows the mean SDDR ( $\frac{suprathreshold\;distortions}{distortion@threshold}$ ) for each of the four types of distortions with low and high distortion levels indicated on the upper *x*-axis. Gray, black, and red symbols refer to YNH-, ON1-, and ON3- listeners, respectively. The circles and diamonds represent data measured in the LRL and the large room. The dashed horizontal line with *y* =1 represents the case where the amount of suprathreshold distortions equals the mean distortion detection thresholds, while the green and red highlighted areas indicate a ratio >1 (above threshold) and <1 (below threshold), respectively. Panels B–E show the relationship between the SDDR and the quality scores for YNH, ON1, and ON3 listeners. Circles and squares refer to the LRL and the large room. The error bars represent one standard deviation.

In the following sections, the means and the standard deviations (error bars indicate one inter-individual standard deviation) of the distortion detection thresholds and quality ratings calculated from individual results of 12 ON1 and 12 ON3 listeners are shown as black and red symbols in Figures 2–5. In addition to this data, data collected in Biberger and Ewert (2022) from young NH listeners (YNH) is replotted in gray symbols to compare the performance of older HI listeners with that of young NH listeners. The YNH data was also included in the statistical analysis reported below. It should be noted that the rooms LRL (used in this study) and Small (used in Biberger & Ewert, 2022), representing rooms with mild reverberation, are not identical, but perceptually very similar.

### Detection Thresholds

Figure 2 shows the mean and standard deviation of the individual detection thresholds for ON1 and ON3 listeners as black and red symbols in the four panels representing different types of distortions. The gray symbols indicate data measured in Biberger and Ewert (2022) with YNH listeners. The abscissa represents the room configurations LRL (mild reverberation), LRL,2M_sep_ (mild reverberation plus two spatially separated maskers), and Large (moderate reverberation).

The Pearson-Correlation Coefficient (PCC) was used as an indicator for the test–retest reliability of the measured distortion detection thresholds calculated from N1 and N3 listeners. PCCs above 0.85 were observed for spectral ripples (PCC: 0.89), nonlinear saturation (PCC: 0.87), and intensity (PCC: 0.88), while a rather low PCC of 0.62 was observed for spatial distortions. The low PCC can be partly explained as a result of ceiling effects observed in the condition where the target speech was presented with two maskers.

For both listener groups and for all types of distortions, substantially higher detection thresholds were measured in the room with maskers (LRL,2M_sep_) than in the rooms without maskers (LRL and Large). In contrast, distortion thresholds measured in the rooms LRL and Large with different amounts of reverberation show clearly smaller differences.

For speech with spectral ripples and intensity distortions presented without maskers (LRL and Large), ON1 and ON3 listeners showed similar distortion detection thresholds, while substantially higher thresholds can be observed for ON3 than for ON1 listeners with maskers (LRL,2M_sep_). In all room configurations, ON3 listeners had substantially higher thresholds for speech distorted by nonlinear saturation than ON1 listeners. A similar, but less pronounced pattern was also observed for speech with spatial position offsets.

A comparison with the YNH data measured in Biberger and Ewert (2022) showed that both HI groups measured in this study had higher distortion detection thresholds than YNH listeners, whereas only slight differences were observed for Spectral ripples and Intensity.

Applying mixed-design 2-way, repeated-measures ANOVA [*room* (LRL, LRL,2M_sep_, Large), *hearing loss* (YNH, ON1, ON3)] to Box-Cox transformed data of each of the four types of distortions always showed a significant effect of the factor *room* [Spectral ripples: *F*(2, 74)=87.5, *p*<.001 (BTC: λ=−0.5); Nonlinear saturation: *F*(2, 74)=73.4, *p*<.001 (BTC: λ=−0.19); Intensity: *F*(2, 74)=122 *p*<.001 (BTC: λ=0.65); Spatial: *F*(2, 74)=53, *p*<.001 (BTC: λ=0.38)] on distortion detection thresholds. Likewise a significant effect of the *hearing loss* was found for Spectral ripples: *F*(2, 37)=16.3, *p*<.001, Nonlinear saturation: *F*(2, 37)=106, *p*<.001, Intensity: *F*(2, 37)=9.3, *p*<.001, and Spatial: *F*(2,37)=43.8, *p*<.001. For spatial distortions, the assumption of equal error variances across groups (Levene's test) was violated despite the Box-Cox transformation in condition LRL,2M_sep_, which increased the risk of a Type 1 error. Given that the reported effect for spatial distortions is highly significant, the risk of a Type 1 error seems rather low. A significant interaction between *room* × *hearing loss* was found for all distortions [Spectral ripples: *F*(4, 74)=3.2, *p*=.018; Nonlinear saturation: *F*(2, 74)=4.3, *p*=.004; Intensity: *F*(4, 74)=4.7, *p*=.002; Spatial: *F*(4, 74)=6.4, *p*<.001].

Taken together, post-hoc pair-wise comparisons (indicated by asterisks in Figure 2) show that in the condition with maskers (LRL,2M_sep_), ON3 listeners consistently performed worse than ON1 listeners. Such behavior is not observed for the conditions without maskers. For both listener groups, significantly higher distortion detection thresholds were found in conditions with than without maskers. Reverberation had only a minor effect on listeners’ detection thresholds. The sensitivity to nonlinear distortions was reduced with increasing HL as indicated by higher thresholds for ON3 than for ON1, and by consistently higher thresholds for HI listeners than for YNH listeners.

### Suprathreshold Quality Ratings

Listeners’ individual scores were averaged across test and retest. The average test–retest PCC within the sub-experiments *Effect of room* and *Effect of masker* was 0.8 and 0.77 for ON1 data and 0.68 and 0.6 for ON3 data. An overview of the test–retest PCCs for each of the 24 participants is provided in Table 3.

**Table 3.** Individual Test–Retest PCC for Each of the 24 Listeners.

Listener	Category	“Rooms”	“Maskers”	Overall
#1	ON1	0.63	0.62	0.6
#2	ON1	0.86	0.81	0.83
#3	ON1	0.71	0.8	0.74
#4	ON1	0.82	0.53	0.62
#5	ON1	0.85	0.58	0.81
#6	ON1	0.82	0.83	0.81
#7	ON1	0.9	0.88	0.86
#8	ON1	0.76	0.78	0.78
#9	ON1	0.84	0.82	0.82
#10	ON1	0.78	0.77	0.73
#11	ON1	0.81	0.9	0.84
#12	ON1	0.82	0.94	0.85
#13	ON3	0.78	0.44	0.4
#14	ON3	0.53	0.46	0.54
#15	ON3	0.82	0.04	0.63
#16	ON3	0.86	0.77	0.81
#17	ON3	0.77	0.82	0.79
#18	ON3	0.48	0.84	0.77
#19	ON3	0.3	0.64	0.52
#20	ON3	0.76	0.66	0.81
#21	ON3	0.73	0.79	0.77
#22	ON3	0.79	0.2	0.59
#23	ON3	0.65	0.75	0.72
#24	ON3	0.72	0.82	0.74

The first and second columns refer to the listener and the corresponding category of hearing loss, while the third and fourth columns refer to PCC Scores based on audio quality ratings for the experiments *Effect of Room* and *Effect of Masker Configuration*, respectively. The last column shows the overall PCC for both experiments.

#### Effect of Room

In Figure 3, the mean and standard deviation of the individual subjective quality scores of ON1 and ON3 listeners for speech (upper panel) and noise (lower panel) subjected to spatial, nonlinear, spectral, and intensity distortions are shown as black and red symbols. Quality scores measured in the LRL and Large room are indicated by circles and diamonds, respectively. The ordinate shows the quality scores, ranging from 0 (“very strong difference”) to 100 (“no difference”). The abscissa indicates the hidden reference, anchor, and each of the four distortions with low and high amounts of distortion.

A difference of about 22 points between listeners’ ratings for low and highly distorted signals (both speech and pink noise) can be observed for the ON1 listeners, while ON3 listeners show an average difference of about 18 points. ON1 listeners always assigned the lowest quality scores to the anchor signal, which was not always the case for the ON3 listeners. For speech, the hidden reference was in 38 and 36 out of 48 cases correctly identified by the ON1 and ON3 listeners, while both ON1 (26 out of 48 correct identifications) and ON3 (25 out of 48 correct identifications) listeners had more difficulties in identifying the hidden pink noise reference.

A mixed-design 5-way, repeated-measures ANOVA [*stimulus* (speech, pink noise), *room* (LRL, Large), *type of distortions* (spatial, nonlinear, spectral, intensity), *distortion level* (low, high), *hearing loss* (YNH, ON1, ON3)] applied to Box-Cox transformed data (λ=3) showed a significant main effect of the factors *room*, *F*(1, 37)=16, *p*<.001, *type of distortions*, *F*(2, 73.7)=34.5, *p* <.001, and *distortion level*, *F*(1, 37)=254.4, *p*<.001, on quality ratings, while no significant effect was found for *stimulus*, *F*(1, 37)=0.01, *p*=.9. A small, significant effect was found for the *hearing loss*, *F*(2, 37)=3.4, *p*=.045.

Bonferroni-corrected pairwise comparisons revealed, with exception of nonlinearly distorted noise in the large room, significant rating differences between low and high distortions for ON1 listeners. For ON3 listeners such significant differences were only observed in 13 out of 16 cases. The Bonferroni-corrected pairwise comparisons of quality ratings of ON1 listeners for speech (upper panel of Figure 3) also revealed that spatial distortions were significantly influenced by the factor *room*, while no room-dependent rating differences were observed for the other types of distortions. The same analysis showed significantly higher quality ratings of ON3 listeners for speech with spectral ripples in the LRL (Low: 81, High: 72) than in the Large room (Low: 72, High: 52) room. The lower panel of Figure 3 shows that both ON1 and ON3 listeners assigned significantly higher scores to noise with high nonlinear distortions and high spatial distortions presented in the Large room than in the LRL. For noise with high intensity distortions, significantly higher quality ratings were obtained from ON3 listeners in the Large (High: 58) than in the LRL (High: 46).

Given the focus on the effects of room and hearing loss in the current study, only significant interactions including these factors are reported in the following. Significant two-way interactions between the factors *room* × *stimulus*, *F*(1, 37)=12, *p* <.001, *room* × *type of distortions*, *F*(2.3, 83.9)=22.2, *p* <.001, and *room* × *distortion level*, *F*(1, 37)=10.5, *p* <.01, and a significant three-way interaction between *room* × *type of distortion* × *stimulus*, *F*(2.3, 84.2)=10.1, *p* <.001, were found. Moreover, significant interactions were found between *hearing loss* × *type of distortions F*(3.9, 73.7)=19.1, *p*<.001, and between *hearing loss* × *type of distortions* × *distortion level*, *F*(5.1, 94)=4.1, *p*=.002. It has to be mentioned that according to the Levene-test, the error variances across groups were not equal for all conditions. Given that the reported interaction effects for *hearing loss* are clearly significant, the risk of a Type 1 error seems rather low.

To summarize, the room had a significant effect on quality ratings, depending on the type of distortion: For spatially distorted speech and noise signals, ON1 and ON3 listeners assigned higher ratings (less perceived distortions) to the Large room with stronger reverberation than in the LRL. Conversely, ON3 listeners rated spectrally distorted speech in the Large room significantly lower than in the LRL, implying that reverberation might also pronounce distortions. Only a slight significant effect of *hearing loss* on quality ratings was observed, whereas a significant interaction indicates that audio quality for different types of distortions is differently perceived depending on the HL.

#### Effect of Masker Configuration

Figure 4 shows average subjective quality scores and interindividual standard deviations from ratings of ON1 (black circles) and ON3 (red circles) for speech with spectral ripples, nonlinear saturation, intensity, and spatial distortions in the LRL as a function of the masker configuration. Gray symbols represent data measured in Biberger and Ewert (2022) with YNH listeners. It should be noted that the parameters for low and high distortions were identical for ON1 and ON3 listeners, but partly differed from those for YNH listeners (see Sec. Methods).

For both HI groups, the hidden reference without maskers (Ref 0M) always received the highest ratings (ON1: 98.4, ON3: 99.5), while lower quality ratings for the hidden reference with two spatially separated maskers, Ref 2M_sep_, of 88.8 and 75 points were obtained for ON1 and ON3, respectively. ON1 listeners assigned average quality ratings of 38.1 and 44.1 to the anchor signal without and with maskers, while ON3 listeners assigned higher quality scores of 61.4 and 50.6, respectively. Thus, ON3 listeners assigned lower ratings to the anchor with maskers than without maskers, opposite to what was observed for the ON1 listeners.

A design, 4-way, repeated-measures ANOVA [*type of distortions* (spatial, nonlinear, spectral, intensity), *distortion level* (low, high), *masker* (0M, 2M_co_, 2M_sep_), *hearing loss* (YNH, ON1, ON3)] applied to Box-Cox transformed data (λ=4.56) showed a significant effect of *type of distortion*, *F*(3, 111)=8.3, *p* < .001, *distortion level*, *F*(1, 37)=131.9, *p* < .001, and *masker*, *F*(1.3, 46.3)=5.9, *p* = .014, on quality ratings, while no significant effect of *hearing loss*, *F*(2,37)=2.8, *p*=.073, was found. In the following, only significant interactions including *masker* and *hearing loss* are reported. Significant two-way interactions between *masker* × *type of distortions*, *F*(4.6, 168.6)=15.3, *p*=<.001, *masker* × *distortion level*, *F*(2, 74)=12.4, *p*=<.001, and *hearing loss* × *masker*, *F*(2.5, 46.3)=4.4, *p*=0.012, were found. The latter-mentioned significant interaction indicates that the masker configuration (0M, 2M_co_, and 2M_sep_) had a significant effect on the listeners’ quality ratings, depending on whether the listeners had normal hearing, a very mild, or moderate HL. Additionally significant three-way interactions were found between *masker* × *type of distortion* × *distortion level, F*(6, 12)=3.3, *p*<.01, and between *masker* × *type of distortion* × *hearing loss, F*(9.1, 168.6)=6.8, *p*<.001. It has to be mentioned that despite the Box-Cox transformation, the Levene test did not show equal error variances across groups (factor *hearing loss*) in all of the considered conditions, which increases the risk of a Type 1 error. Given that the reported effects for *hearing loss* are clearly significant, the risk of a Type 1 error seems rather low. For both listener groups with HL, Bonferroni-corrected pairwise comparisons did not find any significant difference in quality scores between the considered masker configurations 2M_co_ and 2M_sep_. Nevertheless, some interesting (but non-significant) observations are reported in the following. Speech with high spectral ripples, nonlinear saturation, and spatial distortions in the presence of maskers obtained higher ratings from ON1 listeners than without maskers. An opposing effect can be observed for low intensity distortions, where ON1 listeners assigned higher scores to the condition without masker than to that with masker. Similarly, ON3 listeners assigned higher quality scores to nearly all conditions where the distorted speech was presented without maskers than for the conditions with maskers.

In summary, the presence of maskers had a larger and mostly opposite influence on the quality ratings for ON3 than for ON1 and YNH listeners: While the presence of maskers tended to increase quality ratings for ON1 and YNH listeners, it tended to decrease quality ratings for ON3 listeners. For both HI groups, no substantial effect on quality ratings was observed when the maskers were either presented spatially co-located to or spatially separated from the distorted speech.

### Detection Threshold Performance and Audio Quality Ratings

Given that for practical reasons, the parameters for low and high distortions used in the audio quality rating experiment were based on a pilot experiment with two ON1 and two ON3 listeners, it could not be excluded that the assumed suprathreshold distortions were in fact near or below the individual detection threshold for some listeners. The ratio of the applied suprathreshold distortion to the individual distortion detection threshold, $\frac{suprathreshold\;distortion}{distortion@threshold},$ (in the following referred to as SDDR, signal distortion-to-detection ratio) can be used as an indicator for the listener's ability to resolve the distortions based on the individual distortion thresholds. A ratio >> 1 would imply that signal distortions are substantially higher than the distortion threshold, and thus clearly perceived by the listeners, resulting in lower quality ratings. A ratio < 1 would imply that signal distortions were below the individual distortion detection threshold, and were therefore not or hardly perceived by the listeners, resulting in quality scores at 100. An SDDR of 1 indicates that the amount of distortion was at the threshold.

Figure 5A shows the average SDDR for each listener group for the four types of distortions with low and high distortion levels as indicated on the upper *x*-axis. Gray, black, and red symbols represent YNH, ON1, and ON3 listeners, whereas circles and diamonds refer to the LRL and Large room. Since this study used different distortion levels for low and high distortions than those used in Biberger and Ewert (2022) for YNH listeners, the SDDR is also useful with respect to the comparison of the three listener groups. Given that the numerator in the SDDR is identical for each HI listener, the ratio is only driven by individual distortion detection thresholds. For low spatial, nonlinear, and intensity distortions, the mean ratios of several ON1 and ON3 listeners are lower or close to 1 (Spatial: 3 ON1 listeners, 2 ON3 listeners; Nonlinear: 2 ON1, 2 ON3; Intensity: 5 ON1, 5 ON3) indicating that the distortions were in fact often below the detection thresholds and thus not suprathreshold. Given that measured distortion detection thresholds represent the distortion levels at which listeners had a correct rate of 71% (for identifying the interval with the distorted sound), an SDDR below 1 does not strictly mean that listeners cannot perceive the distortion. With the exception of high nonlinear distortions applied to ON3 listeners, the SDDR is above 1 for all other high distortions in each of the HI listener groups, and thus is expected to be perceptible within the quality rating experiments. For YNH listeners, the SDDR is in most cases well above 1. Panels B–E of Figure 5 show the relationship between suprathreshold quality ratings (shown on the *x*-axis) and the SDDR (shown on the *y*-axis) of YNH, ON1, and ON3 listener groups for spatial, nonlinear saturation, spectral ripples, and intensity distortions. Circles and diamonds represent LRL and the Large room. For most cases, it can be observed that listener groups who obtained large SDDR assigned lower quality scores, whereas groups with small SDDR assigned higher quality scores. Reasonably high PCCs shown in panels B-E indicate a strong linear relationship between SDDR and quality scores.

## Discussion

### Influence of CAEs on Distortion Detection Thresholds Depends on Hearing Loss

In agreement with previous psychoacoustic studies, higher distortion detection thresholds were observed in the presence of maskers (LRL,2M_sep_) compared to conditions without maskers. Figure 2 clearly shows that ON3 listeners had consistently more difficulties when the distorted speech was presented with maskers than ON1 listeners. Such consistent behavior was not observed without maskers, e.g., for spectral ripples or intensity distortions, where ON1 and ON3 listeners obtained similar distortion detection thresholds. For these distortions in situations without maskers, it appears that the HL compensation strategy enabled performance similar to that of YNH listeners, while it did not help much in situations where it was required to separate the acoustic information of the distorted target and interfering sound sources (e.g., Kochkin, 2000).

Reverberation had only a minor effect on distortion detection thresholds in rooms LRL (T60: 0.5 s) and Large (T60: 1.5 s) for both HI groups. While Biberger and Ewert (2022) found significant threshold differences for YNH listeners between anechoic and the Large room (same as used in this study) for Spectral ripples, Nonlinear saturation, and Spatial distortions, differences between Small (similar to LRL) and the Large room were only found for Spatial distortions. Thus, stronger reverberation effects may have emerged in this study had an anechoic condition been included for comparison with the Large room.

Although the HL compensation strategy is expected to restore audibility, reduced frequency selectivity and loss of cochlear compression likely affect the internal sound representation. This may explain the reduced sensitivity to nonlinear saturation distortions with increasing HL across all room conditions. According to the Generalized Power Spectrum Model (Biberger & Ewert, 2016), the largest differences in power and envelope power SNRs between nonlinearly distorted and unprocessed speech occurred between 2 kHz and 3.15 kHz. Impaired frequency resolution and elevated internal noise (e.g., Kollmeier, 1999) likely contributed to HI listeners’ difficulties in detecting distortion components.

Intensity JNDs in the LRL were comparable across groups (YNH: 1.4 dB; ON1: 1.8 dB; ON3: 2.1 dB), aligning with previous findings, e.g., Whitmer and Akeroyd (2011), who reported no substantial JNDs between NH (1.8 dB) and aided HI listeners (2.2 dB). Although reduced cochlear compression in HI listeners might lead to steeper loudness growth (and thus lower JNDs), factors such as internal noise (e.g., Kollmeier, 1999), reduced temporal and spectral resolution may counteract this effect.

Similarly, spectral ripple detection thresholds were comparable between HI groups (ON1: 8.4 dB, ON3: 8.3 dB) in speech without maskers, implying effective compensation by NAL-R. Despite broader auditory filters expected in ON3, reduced spectral resolution had only a minimal impact. The small threshold differences (∼2 dB) between YNH and HI groups are interesting, given that spectral ripples are common in everyday scenarios, such as in hearing devices where the direct sound typically interferes with the delayed processed signal (see Denk et al., 2021; Ohlmann et al., 2024; Stone et al., 2008) or reverberant environments.

In this study, azimuth JNDs increased with HL (YNH: 2°, ON1: 14°, ON3: 17° in the LRL). These are higher than values reported by Häusler et al. (1983) for broadband noise in anechoic conditions (<6°) for HI listeners, likely due to the reverberant environments and different stimuli used in the current study.

### Suprathreshold Quality Ratings

The overall differences in the quality rating patterns found for the YNH and HI groups imply that HI listeners perceive distorted signals differently from YNH listeners. In line with literature, loudness perception of intensity distorted signals is more similar between groups of HI listeners (ON1 and ON3) than between YNH and HI listeners. From this perspective, e.g., the poorer ability in HI than in NH listeners to discriminate natural soundscapes as found in Miller-Viacava et al. (2024) seems plausible.

#### Hearing Loss Effects the Consistency of Quality Ratings

As shown in Table 3, ON1 listeners with very mild HL exhibited higher test–retest reliability (*Effect of room*: 0.8, *Effect of masker*: 0.77) than ON3 listeners with moderate HL (*Effect of room*: 0.68, *Effect of masker*: 0.6). Both groups, however, showed lower test–retest reliability than YNH listeners in Biberger and Ewert (2022) who achieved mean PCCs of 0.91 and 0.95 for *Effect of room* and *Effect of masker*, respectively. Similar reductions in reliability for HI listeners were reported by Tan et al. (2003) and Tan and Moore (2008), where YNH- and HI-listeners rated audio quality for nonlinearly distorted signals. They mentioned two potential reasons for such reduced consistency: (a) HI listeners may be less sensitive to nonlinear distortions than YNH listeners. (b) For HI listeners, the undistorted reference signal may also sound somewhat distorted, which might result in less consistent ratings. Additionally, Narendran and Humes (2003) and Gabrielson et al. (1988) found higher test–retest reliability in younger than in older HI-listeners (see Table 2 in Narendran & Humes, 2003), suggesting age may affect quality rating consistency. However, since the ON1 and ON3 groups were age-matched in this study, the reduced test–retest reliability in the current HI listeners is more likely due to HL rather than age.

#### Effect of Room Depends on Type of Distortion

Limitations in the auditory system determine whether processed signals are perceived as acoustically transparent. In this regard, consequences of room reverberation on audio quality perception, where (late) sound reflections may partly mask distorted parts of the signals, are interesting for many applications, such as sound reproduction systems. In this study, reverberation affected quality ratings in both HI groups, such as signals with spatial position offsets of 30° azimuth received higher ratings in the Large room (T60: 1.5 s) than in the LRL (T60: 0.5 s), indicating reduced spatial resolution in more reverberant environments. Such a pattern was also observed for YNH in Biberger and Ewert (2022) and a similar effect was reported in Brinkmann et al. (2017) for YNH listeners, where reverberation enhanced perceived authenticity. An opposing effect, where room reflections can intensify signal distortions, was observed in speech with spectral ripples, where ON3 listeners rated the quality of speech with spectral ripples significantly lower in the Large room than in the LRL. This could be the result of an interaction between mild low-frequency HL in ON3 listeners and low-frequency room modes, which may facilitate the perception of signal distortions.

#### Effect of Masker Depends on Hearing Loss

As shown in Figure 4, ON1 listeners, similar to YNH in Biberger and Ewert (2022), often rated distorted signals higher when maskers were present, likely due to partial masking of distortions. In contrast, ON3 listeners consistently assigned lower quality scores to masked conditions, and even rated undistorted references with maskers (Ref 2M_sep_) lower than distorted signals without maskers, suggesting greater difficulty in separating the target from the masker. Despite these rating differences, average responses to the post-experiment questionnaire, where the listeners had to indicate on a Likert-scale “How difficult was it to separate the target from the masker?” were similar for ON1 and ON3 (Likert scores of 0.33 and 0.17), indicating subjective difficulty ratings were not group-dependent. Prior research (e.g., Bertoli et al., 2005; Carroll et al., 2016; Legér et al., 2012; Pichora-Fuller et al., 1995; Pichora-Fuller & Souza, 2003) has reported age-related effects on suprathreshold auditory processing or cognitive factors in challenging CAEs with maskers and reverberation. Thus, age is likely a contributing factor when comparing ON1, and ON3 results with YNH, particularly under masked conditions.

In conclusion, ON1 and ON3 listeners showed similar quality ratings in conditions without maskers (see Figure 3), but different ratings with maskers (see Figure 4). This is consistent with findings in psychoacoustics or speech intelligibility where listeners had primarily difficulties in situations with maskers (Buchholz et al., 2023; Mansour et al., 2021; Plomp, 1978).

### Predictability of Distortion Thresholds and Audio Quality

Previous studies have reported correlations between listeners’ individual PTAs and data in speech perception or psychoacoustics (e.g., George et al., 2007; Strelcyk & Dau, 2009). In this study, PTA_500Hz−4kHz_ was calculated as the average of the participant's best ear over octave frequencies 0.5, 1.0, 2.0, and 4.0 kHz and used to examine potential degradation beyond audibility under NAL-R amplification. As shown in Table 4, significant correlations between the individual PTAs_500Hz−4kHz_ and distortion detection thresholds were found primarily in masker conditions (LRL,2M_sep_) for all distortion types, while in conditions without maskers, significant correlations above 0.5 were only observed for nonlinear saturation. This suggests that despite HL was compensated by NAL-R, increasing hearing loss impairs performance in CAEs. The strong correlation for nonlinear saturation indicates deficits beyond audibility. In contrast to the distortion detection thresholds, no significant correlations were found between PTAs_500Hz−4kHz_ and quality ratings.

**Table 4.** (Statistically Significant) Correlations Between the Pure Tone Average (PTA_500Hz−4kHz_) from the Better ear and Distortion Detection Thresholds.

	LRL	LRL,2M_sep_	Large
Spectral ripples	0.02	0.53**	0.1
Nonlinear saturation	0.59**	0.39*	0.77**
Intensity	0.18	0.56**	0.02
Spatial	0.3	0.49**	0.25

Significance levels of .05 and .01 are reported by * and **, respectively. The abbreviations LRL and Large refer to the living room lab (T60: 0.5 s) and the large room (T60: 1.5 s), while LRL,2M_sep_ refers to the living room lab with two spatially separated maskers.

For applications such as hearing devices, it would be desirable to use individually and rapidly measured distortion thresholds as a predictor for the perception of suprathreshold distortions. High correlations between SDDR (signal distortion-to-detection ratio) and quality ratings were observed for most conditions (except binaural distortions in LRL and intensity distortions in the Large room) as shown in Figure 5B–E. The SDDR, which normalizes the amount of (suprathreshold) signal distortions by the listener's detection threshold, effectively estimates a perceptual distance where larger ratios (>>1) imply suprathreshold distortions and accordingly lower quality ratings. This method might be useful to coarsely estimate suprathreshold quality ratings between groups with different degrees of HL when only distortion detection thresholds were measured.

The calculation of PCCs between listeners’ individual distortion detection thresholds and quality ratings, reported in Table 5, is a more direct way to assess whether distortion thresholds can be used as a predictor for listeners’ quality ratings. As indicated by red symbols in parentheses, in nearly all cases higher correlations between detection thresholds and quality ratings were found for ON3 than for ON1 listeners. Therefore, suprathreshold ratings of ON3 listeners seemed to be more dependent on their threshold performance than in ON1 listeners. Taken together, significant correlations between detection thresholds and quality ratings were mainly observed for nonlinear saturation and intensity distortions, which seemed to be largely driven by ON3 listeners.

**Table 5.** Statistically Significant Correlations Between Distortion Detection Thresholds (Rows) and Quality Ratings (Columns) of All Participants for Spectral Ripples (Lin), Nonlinear Saturation (Nonlin), Intensity (Int), and Spatial (Bin) Distortions.

Thresholds	Ratings	Dist1_LRL_	Dist2_LRL_	Dist1_Large_	Dist2_Large_	Dist1_LRL,2Msep_	Dist2_LRL,2Msep_
Lin_LRL_				*		**(*)	**(*)
Lin_Large_		*(*)		*(*)		*	*
Lin_LRL,2Msep_						**	(*)
Nonlin_LRL_		*(*)			*
Nonlin_Large_		**(**)	*		**(**)		(*)
Nonlin_LRL,2Msep_		**(*)	*	*	**(*)		(*)
Int_LRL_		*				*	*
Int_Large_		*	*	*	*
Int_LRL,2Msep_		**(**)	*	**(*/*)	(**)
Bin_LRL_			*	(*)	(*)
Bin_Large_					(*)
Bin_LRL,2Msep_		*(**)	*(*)		*		

Dist1 and Dist2 refer to low and high distortion levels, while the room condition is shown as subscripts, e.g., Lin_LRL_ or Dist1_LRL_ indicates the room condition LRL. The abbreviations LRL and Large refer to the living room lab and the large room, while LRL,2M_sep_ refers to the living room lab with two spatially separated maskers. Significance levels of .05 and .01 are reported by * and **, respectively. Asterisks shown in parentheses indicate correlations based only on ON1 (black) and ON3 (red) listeners.

## Summary and Conclusions

Distortion detection thresholds and audio quality ratings for distorted speech and pink noise in scenes with varying acoustic complexity were measured for two groups of older listeners with either a very mild (ON1) or moderate (ON3) hearing loss. For both groups, hearing loss was compensated by using NAL-R prescription, and the listeners were allowed to set the stimulus presentation level to a comfortable sound level. The following conclusions can be drawn:

- Similar distortion detection thresholds were often observed for ON1 and ON3 listeners in rooms without maskers. Conversely, in the presence of masker sounds, ON1 listeners had consistently lower distortion detection thresholds than ON3 listeners. This agrees with findings from previous studies in psychoacoustics and speech perception, where increasing hearing loss was often associated with performance degradation in complex acoustics environments with maskers, while only a slight or even no performance reduction was observed in situations without maskers.
- As with the distortion threshold findings, audio quality ratings of ON1 and ON3 listeners are more similar for distorted signals without maskers than for distorted signals with maskers. In comparison to the conditions without maskers, ON1 listeners rated audio quality higher (less distortions perceived) in the presence of maskers, while ON3 listeners consistently assigned lower quality scores. Such group-dependent differences are probably based on the difficulties of ON3 listeners in separating the target from the masker sources.
- Reverberation had only a slight effect on distortion detection thresholds, while the effect of reverberation on quality ratings strongly depended on the type of distortions and signals, e.g., spatial distortions received higher quality ratings in the room with a reverberation time of 1.5 s than in the room with 0.35 s. These findings indicate that inaccurate reproduction of the sound source position is less critical in rooms with strong than in rooms with mild reverberation.
- Distortion detection thresholds and quality ratings found for ON1 and ON3 listeners for spectral ripples and intensity distortions are similar to those from YNH listeners. Thus, it can be expected that these listener groups have similar abilities in resolving differences in spectral coloration. Accordingly, hearing devices aiming at acoustical transparency have to fulfill similar requirements for the considered listener groups regarding spectral coloration. On the other hand, listeners’ sensitivity to nonlinear saturation distortions strongly decreased with increasing hearing loss, and therefore, hearing loss defines the level of tolerable nonlinear distortions of the device or algorithms in order to be perceived as acoustically transparent.
- The better-ear pure tone average (PTA_500Hz−4kHz_) was a good predictor for distortion detection thresholds in conditions with maskers, but not for most of the conditions without maskers. This indicates that the hearing loss compensation in this study, aiming at restoring audibility, works well in situations without maskers, while a compensation strategy beyond restoring audibility would be required to cope with situations where maskers occur.

## Notes

1. It should be noted that this procedure is not free-field equivalent and differs due to minor linear filtering effects. Free-field equivalent presentation could be obtained for other (audiometric) headphone types for which reference equivalent sound pressure level (RETSPL) measurements or other perceptual corrections are available. However, since all signals used in this study are presented well above threshold we considered the comparability of the headphone type to many earlier studies most relevant.
